# Genome Sequencing-Based Mining and Characterization of a Novel Alginate Lyase from *Vibrio alginolyticus* S10 for Specific Production of Disaccharides

**DOI:** 10.3390/md21110564

**Published:** 2023-10-27

**Authors:** Zhiqiang Shu, Gongming Wang, Fang Liu, Yingjiang Xu, Jianan Sun, Yang Hu, Hao Dong, Jian Zhang

**Affiliations:** 1 Department of Food Science and Technology, Shanghai Ocean University, Shanghai 200120, China; s13970318519@163.com; 2 Shandong Marine Resource and Environment Research Institute, Yantai 264006, China; wgmsd105@163.com (G.W.);; 3 Yantai Key Laboratory of Quality and Safety Control and Deep Processing of Marine Food, Yantai 264006, China; 4 Qingdao Key Laboratory of Food Biotechnology, College of Food Science and Engineering, Ocean University of China, Qingdao 266404, China; sunjianan@ouc.edu.cn (J.S.);; 5 Key Laboratory of Biological Processing of Aquatic Products, China National Light Industry, Qingdao 266404, China

**Keywords:** alginate lyase, alginate oligosaccharide, heterologous expression, product specificity, complete genome sequencing

## Abstract

Alginate oligosaccharides prepared by alginate lyases attracted great attention because of their desirable biological activities. However, the hydrolysis products are always a mixture of oligosaccharides with different degrees of polymerization, which increases the production cost because of the following purification procedures. In this study, an alginate lyase, Alg4755, with high product specificity was identified, heterologously expressed, and characterized from *Vibrio alginolyticus* S10, which was isolated from the intestine of sea cucumber. Alg4755 belonged to the PL7 family with two catalytic domains, which was composed of 583 amino acids. Enzymatic characterization results show that the optimal reaction temperature and pH of Alg4755 were 35 °C and 8.0, respectively. Furthermore, Alg4755 was identified to have high thermal and pH stability. Moreover, the final hydrolysis products of sodium alginate catalyzed by Alg4755 were mainly alginate disaccharides with a small amount of alginate trisaccharides. The results demonstrate that alginate lyase Alg4755 could have a broad application prospect because of its high product specificity and desirable catalytic properties.

## 1. Introduction

Alginate is a type of abundant polysaccharide found in marine resources. It is composed of β-D-mannuronic acid (M) and α-L-glucuronic acid (G) linked by 1,4-glycosidic bonds [1]. According to the different forms of the connection between M and G through glycosidic bonds, there are three kinds of chimeras in alginate molecules: homopolymeric M (polyM), homopolymeric G (polyG), and heteropolymeric form (polyMG) [2]. Alginate is usually used as a stabilizer, emulsifier, thickener, and additive in the food, cosmetic, and pharmaceutical industries due to its favorable thickening and gelation characteristics [3,4,5]. However, the high molecular weight of alginate results in its low water solubility and bioavailability. Alginate oligosaccharides (AOS) are the oligomers of alginate with the degrees of polymerization of 2 to 25 [6]. They attracted great attention because of their desirable biological activities, such as anti-tumor [7], anti-inflammatory [8], neuroprotection [9], immunomodulation [10], anti-obesity [11], anti-bacterial [12], antioxidant [13], and anti-diabetic [14] activities. AOS are widely used in food and health care, medical and pharmaceutical [15], agrochemical [16], and other industries, illustrating their wide application prospects.

Current methods for alginate oligosaccharide preparation include physical [17], chemical [18], and enzymatic [19] methods. The commonly used physical methods include radiation [17], thermal degradation [20], and ultrasound [21]. The chemical methods include hydrochloric acid [22] and oxidative degradation [18]. Compared to physical and chemical methods, enzymatic methods offer several advantages in producing AOS [4]. Enzymatic methods mainly use alginate lyase to degrade alginate. It is widely used because of its high efficiency, high specificity, controllability, mild reaction conditions, and environmental friendliness.

Alginate lyases come from a wide range of sources, including marine animals, marine algae, and microorganisms, with microbial sources being the most abundant [23]. Alginate lyases are categorized into 14 polysaccharide lyase families (PL5, 6, 7, 8, 14, 15, 17, 18, 31, 32, 34, 36, 39, and 41) based on the Carbohydrate-Active enZYmes (CAZy) database (http://www.cazy.org/, accessed on 10 April 2023) [24]. Among these families, the PL7 family has the highest number of alginate lyases and is further divided into six subfamilies [25]. Furthermore, alginate lyases can be classified into PM-specific lyases (EC 4.2.2.3), PG-specific lyases (EC 4.2.2.11), and bifunctional lyases that degrade both PM and PG (EC 4.2.2.-) based on their substrate specificity [26]. Additionally, they can be categorized based on their action mode into endo-type lyases that produce oligosaccharides by cleaving the internal glycosidic bonds [27] and exo-type lyases that produce monomers or dimers by gradual degradation from the end of the alginate polymer [28,29]. Although many alginate lyases were identified, most of them degraded alginate into AOS with different degrees of polymerization [30,31]. The downstream procedure for purification of oligosaccharide with a single structural form would significantly increase the production cost, which makes it uneconomic. Moreover, low catalytic performances also restrict the wide use of alginate lyases. Therefore, it is still urgent to screen and characterize novel alginate lyases with desirable catalytic properties, especially those with high product specificity. Gut microorganisms can be regarded as a promising source for the identification of alginate lyases with desirable catalytic performances, because these microorganisms play a key role in digesting algae in marine animals [32].

In this study, we identified a strain named *Vibrio alginolyticus* S10 from the intestine of sea cucumbers with the ability to degrade alginates effectively. We performed complete genome sequencing and further identified an alginate lyase gene *alg4755* by genomic analysis. Furthermore, we overexpressed the alginate lyase Alg4755 in *Escherichia coli* BL21(DE3) and characterized its catalytic properties. Alg4755 exhibited desirable thermal and pH stability, and high product specificity. The main products were identified as alginate disaccharides with a small amount of alginate trisaccharides. Alg4755 shows great potential in the preparation into AOS with specific degrees of polymerization (DP), which should be beneficial to promoting the development of alginate-related industries.

## 2. Results and Discussion

### 2.1. Screening and Identification of Strain S10

**Figure 1:**
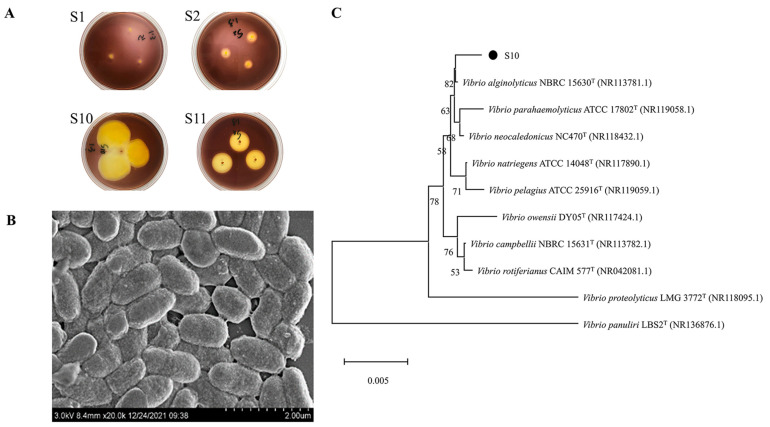
Screening and identification of strain S10. (**A**) Transparent circle of solid culture medium (S1, S2, S10, and S11 are four strains that can degrade sodium alginate screened from the intestinal microorganisms of sea cucumber). (**B**) SEM results of strain S10. (**C**) The neighbor-joining phylogenetic tree of strain S10 based on the 16S rRNA gene sequences. The bootstrap values of each branch were tested by 1000 repetitions.

The microorganisms in the gut of sea cucumber were initially screened with sodium alginate as the only carbon source, and then re-screened by the 3,5-Dinitrosalicylic acid (DNS) method. Four strains with higher activity that could use sodium alginate for growth and metabolism were screened. The strains were stained with Gram’s iodine solution, resulting in the formation of bright yellow hyaline circles (Figure 1A). In this experiment, the enzymatic activity of alginate lyase was proportional to the size of the hyaline circle. The results show that strain S10 was an alginate lyase-secreting bacterium and had higher enzymatic activity compared to the three other alginate lyase-producing strains (S1, S2, and S11).

Strain S10 was identified as a Gram-negative bacterium through Gram staining. Its morphology was observed as short rods with lengths ranging from 0.8 to 1.4 μm and widths ranging from 0.5 to 0.7 μm under a scanning electron microscope (JSM-6380LV, JEOL, Tokyo, Japan) (Figure 1B). The 16S rRNA gene of strain S10 was amplified, sequenced, and used to construct a phylogenetic tree. The results were then analyzed using the EzbioCloud server and referenced to the NCBI database for species identification. The results show >99% identification with members of the Vibrio genus. Furthermore, it had the highest similarity of 99.79% with *V. alginolyticus* NBRC 15630 (Figure 1C). As a result, strain S10 could be identified as V. alginolyticus. The 16S rRNA gene sequence of strain S10 was submitted to NCBI with the accession number of OL944400.1.

So far, only a few alginate lyases were studied from V. alginolyticus, and all of them showed low catalytic properties, such as the alginate lyase from *V. alginolyticus* ATCC 17749 [33] and the alginate lyase Alg62 [34]. Since S10 was proven to degrade alginate efficiently, finding the key alginate lyases with high catalytic performances was hopeful.

### 2.2. Complete Genome Analysis of Strain S10

High-throughput sequencing technology was used to obtain the fundamental information of the complete genome sequence (Sequence Read Archive accession number SRR26253840) of strain S10 (Table S1). The complete length of the S10 genome was 5,397,046 bp with a GC content of 44.59%. The genome comprises two chromosomes and a plasmid. Chromosome 1 has a total length of 3,390,843 bp and a GC content of 44.64% (GenBank accession number CP135968.1). Chromosome 2 has a total length of 1,876,626 bp and a GC content of 44.45% (GenBank accession number CP135969.1). A total of 4936 coding sequences (CDS) representing a total of 4,587,936 bp were successfully predicted using GeneMarkS software [35], which accounted for 85.01% of the complete genome. The average length of CDS was 929.48 bp. The genome also contained 65 tandem repeats with 37,039 bp, which accounted for 0.81% of the complete genome. Through the prediction of non-coding RNAs, it was discovered that strain S10 contained a total of 37 rRNAs, including 12 23S rRNAs, 12 16S rRNAs, and 13 5S rRNAs. Additionally, there were 57 sRNAs and 127 tRNAs. The strain also contained nucleic acid sequences of 12 genomic islands and 4 prophages. To comprehensively demonstrate the characteristics of S10, we used the Cricos [36] software to generate genomic circle maps (Figures S1–S3).

### 2.3. Sequence Analysis of Alg4755

**Figure 2:**
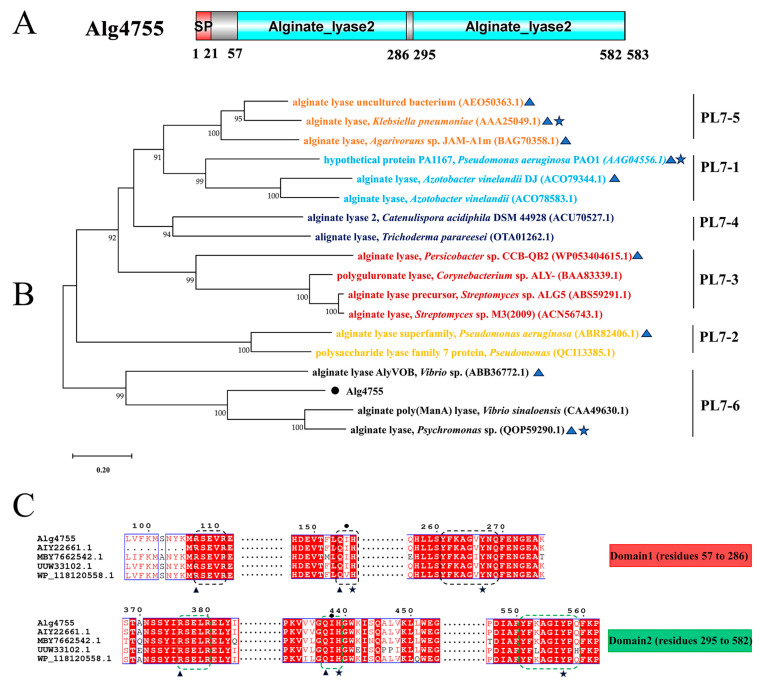
Sequence analysis of Alg4755. (**A**) Schematic diagram of the structural domains of alginate lyase Alg4755. The signal peptide (SP) was predicted using the SignalP 5.0 Server, while the conserved domains were analyzed using the Conserved Domain Database. (**B**) Phylogenetic analysis of Alg4755 with other alginate lyases from the PL7 family using MEGA 11. The bootstrap values of each branch were tested by 1000 repetitions. Alg4755 is marked with a dot. The characterized alginate lyases are marked with triangles. Structure-solved alginate lyases are marked with a five-pointed star. (**C**) Multiple sequence alignment of Alg4755 with related alginate lyases (MBY7662542.1, alginate lyase from V. atlanticus; UUW33102.1, alginate lyase from Vibrio sp.; AIY22661.1, alginate lyase from Vibrio sp. W13; WP_118120558.1, and alginate lyase from Vibrio sp. dhg). The conserved sequences of the PL7 family catalytic domains are marked with black boxes (N-terminal, Domain1) and green boxes (C-terminal, Domain2). Black triangles indicate neutralizing residues. Black stars indicate catalytic acids/bases. Amino acids below the black circles indicate residues associated with catalytic substrates.

Through genome mining, three alginate lyase genes were identified in strain S10, namely alg4755, alg4756, and alg4760. Homology analysis results show that the sequence similarity between alg4755 and alg4756 is 12.29%, and the sequence similarity between alg4755 and alg4760 is 8.23%. Among these genes, alg4755 stood out due to its possession of two catalytic active domains, which is a relatively rare occurrence [37]. This led us to hypothesize that alg4755 may exhibit higher activity or unique product characteristics. Consequently, we selected this enzyme for further investigation. The alg4755 encoded a putative alginate lyase of 583 amino acids with a predicted signal peptide of 21 amino acids. Furthermore, the theoretical molecular weight (MW) and isoelectric point (PI) of Alg4755 were 66.2 kDa and 4.97, respectively. The alginate lyase Alg4755 was highly homologous to the polysaccharide lyase family 7 proteins WP_053349965.1 and WP_250155038.1. However, there are at least 100 entries in GenBank of alginate lyases with >90% similarity to Alg4755 were not heterologously expressed and characterized. Alg4755 was classified in the polysaccharide lyase family 7 (PL7) based on sequence comparison. According to the comparison with the NCBI Conserved Domain Database, Alg4755 was predicted to have two catalytic domains (Figure 2A). This is relatively rare among PL7 alginate lyases [38]. A further sequence alignment of Alg4755 with other PL7 alginate lyases indicated that it belonged to subfamily 6 of the PL7 family (Figure 2B). Moreover, the two catalytic domains of Alg4755 both had the conserved motifs (R*E*R, Q(I/V)H, Y*KAG*Y*Q) in PL7 family alginate lyases (Figure 2C) [26,39]. The amino acid sequence of Alg4755 was submitted to NCBI with the accession number of OQ884715.1.

### 2.4. Expression and Purification of Alginate Lyases Alg4755

**Figure 3:**
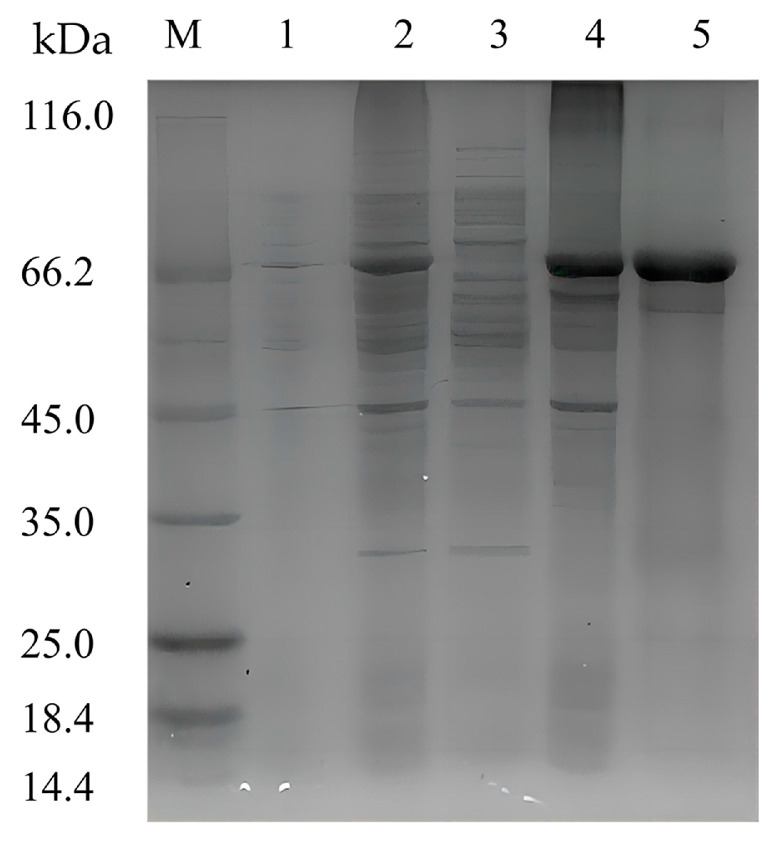
SDS-PAGE analysis of Alg4755. Lane M, molecular weight marker (Solarbio, Beijing, China); Lane 1, uninduced cell lysate; Lane 2, induced cell lysate; Lane 3, supernatant of induced cell lysate; Lane 4, pellet of induced cell lysate; and Lane 5, purified Alg4755 from the pellet of induced cell lysate.

To characterize the recombinant enzyme, the alg4755 gene was cloned into the expression vector pET-28a (+) and transformed into *E. coli* BL21(DE3). The recombinant strain DE4755 containing the alg4755 gene was induced by isopropyl-β-D-1thiogalactopyranoside (IPTG) to produce alginate lyase. After purification by Ni-agarose resin affinity chromatography, the results were analyzed by sodium dodecyl sulfate polyacrylamide gel electrophoresis (SDS-PAGE) (Figure 3). A single band of about 66 kDa was observed, which was consistent with the predicted MW. It was worth noting that most of Alg4755 was expressed in the form of inclusion bodies with alginate lyase activities, which were used for further experiments. After purification, the specific activity of Alg4755 was determined as 961.94 U/mg with the purity increasing nearly 8.2 times (Table 1). Compared with other alginate lyases from V. alginolyticus, Alg4755 had higher enzymatic activity. For example, the specific activity of alginate lyase Alg62 from *V. alginolyticus* was 888.4 U/mg [34], and the specific activity of alginate lyase from *V. alginolyticus* ATCC 17749 was 1.93 U/mg [33].

### 2.5. Characterization of Alg4755

**Figure 4:**
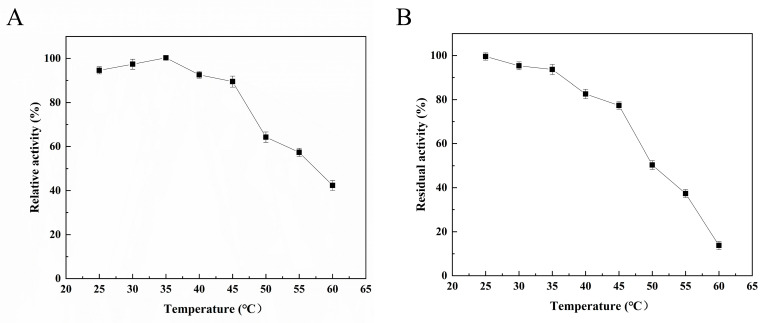
Effect of temperature on Alg4755 activity. (**A**) The optimal temperature of Alg4755. Reactions were conducted in 50 mM Tris–HCl buffer (pH 8.0) at different temperatures for 30 min. (**B**) Thermal stability of Alg4755. The residual activity of Alg4755 was measured after preincubation at different temperatures for 2 h in 50 mM Tris-HCl buffer (pH 8.0). The highest activity was set to 100%. Each value represents the mean of three replicates ± standard deviations.

The enzymatic properties of Alg4755 were further characterized. The optimal reaction temperature of Alg4755 was 35 °C (Figure 4A), and it retained more than 60% activity between 25 °C and 50 °C, illustrating its wide temperature adaptability. To assess the thermal stability of the recombinant Alg4755, residual activity was measured after incubating at different temperatures for 2 h. The results show that the Alg4755 maintained over 50% of its initial enzymatic activity when incubated at temperatures ranging from 25° C to 50 °C for 2 h (Figure 4B). Compared with one other alginate lyase, Alg4755 showed higher thermal stability. For example, the optimal temperature of Alg4755 was similar to that of the alginate lyase AlgB from Vibrio sp. Ni1 but was more thermally stable [40]. The higher thermal stability makes Alg4755 more suitable for harsh reaction conditions.

**Figure 5:**
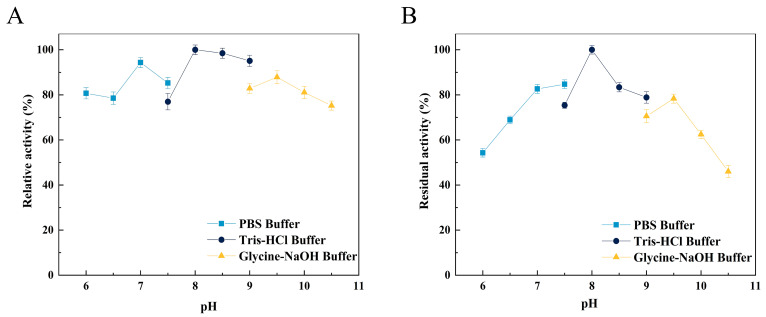
Effect of pH on the activity of Alg4755. (**A**) The optimal pH of Alg4755. The reactions were carried out at 35 °C for 30 min in different buffers of pH 6 to 10.5. (**B**) The pH stability of Alg4755. After incubation of Alg4755 in buffers of different pH (6–10.5) for 2 h at 35 °C, residual enzyme activity was measured. The highest activity was set to 100%. Each value represents the mean of three replicates ± standard deviations.

The optimal pH of Alg4755 was 8.0 (Figure 5A), as well as retaining more than 50% initial activity at pH ranging from 6.0 to 10.0 after incubation for 2 h (Figure 5B). Alg4755 had higher activity at alkaline conditions, which indicated that Alg4755 was an alkaline alginate lyase. In previous studies, most of the reported alginate lyases showed optimal pH and stability in the neutral environment [31]. Similar to Alg4755, Aly08 from Vibrio sp. SY01 also had higher activity and stability in alkaline conditions [41]. Furthermore, Alg4755 could maintain desirable residual activity at a wide range of pH.

**Figure 6:**
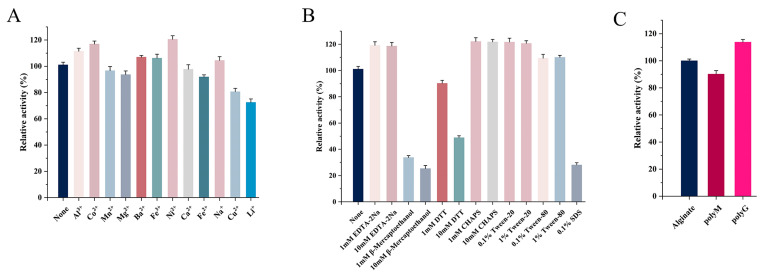
(**A**) Effect of different metal ions on the activity of Alg4755. (**B**) Effect of different concentrations of inhibitors and detergents on the activity of Alg4755. The enzymatic activity without other reagents served as the control, and the enzymatic activity was designated as 100%. (**C**) Substrate specificity of Alg4755. The activity towards sodium alginate was determined as the 100% relative activity. Each value represents the mean of three replicates ± standard deviations.

The effects of metal ions (1 mM) on Alg4755 activity are shown in Figure 6A. Al^3+^, Co^2+^, Ba^2+^, Fe^3+^, and Ni^2+^ had different degrees of promotive effects. Previous studies showed that many metal ions could enhance the enzymatic activity. For instance, the activity of AlgNJ04 increased to 136.2% and 125.32% after activation with 1 mM K^+^ and Ca^2+^, respectively [31]. Furthermore, 1 mM Mn^2+^ and Co^2+^ were found to significantly improve the enzymatic activity of Alyw201 [42]. However, Fe^2+^, Cu^2+^, and Li^+^ had inhibitory effects on the Alg4755 activity, while other metal ions had almost no effect on the enzymatic activity. Moreover, Alg4755 maintained more than 60% relative activity after reacting with various metal ions, illustrating its high resistance toward metal ions. The effect of different concentrations of inhibitors and detergents on the Alg4755 activity is shown in Figure 6B. β-mercaptoethanol, DTT, and SDS all inhibited the Alg4755 activity, which might be ascribed to the conformational change. With the concentration increasing from 1 to 10 mM, the inhibition effect was more obvious. However, EDTA-2Na, Tween-20, Tween-80, and 3-[(3-Cholamidopropyl) dimethylammonio]-1-propanesulfonate (CHAPS) had an activation effect on Alg4755 activity.

### 2.6. Substrate Specificity and Kinetic Parameters of Alg4755

We investigated the substrate specificity of Alg4755 using sodium alginate, polyM, and polyG as substrates. The results show that Alg4755 could degrade these three substrates, which indicated that Alg4755 was a bifunctional alginate lyase (Figure 6C). Alg4755 preferred to degrade polyG compared to polyM. This may be related to its conserved region (QIH). Several studies reported that enzymes responsible for degrading G blocks and M blocks contain conserved regions with QIH and QVH amino acid residues, respectively. This suggested that the amino acid residue I might specifically recognize the polyG block [39]. In addition, we also determined the kinetic parameters of Alg4755 toward sodium alginate, polyM, and polyG (Table 2). The K_m_ values of Alg4755 with sodium alginate, polyM, and polyG as substrates were 5.41 mM, 3.64 mM, and 1.28 mM, respectively. Alg4755 had a lower K_m_ value for polyG, indicating that Alg4755 had a higher affinity for the G block than for the M block. The k_cat_ values of Alg4755 for sodium alginate, polyM, and polyG are 3.82 s^−1^, 7.09 s^−1^, and 20.55 s^−1^, respectively. It showed that the catalytic efficiency of Alg4755 to G block was higher than that to M block.

### 2.7. Analysis of the Degradation Products and Action Pattern of Alg4755

**Figure 7:**
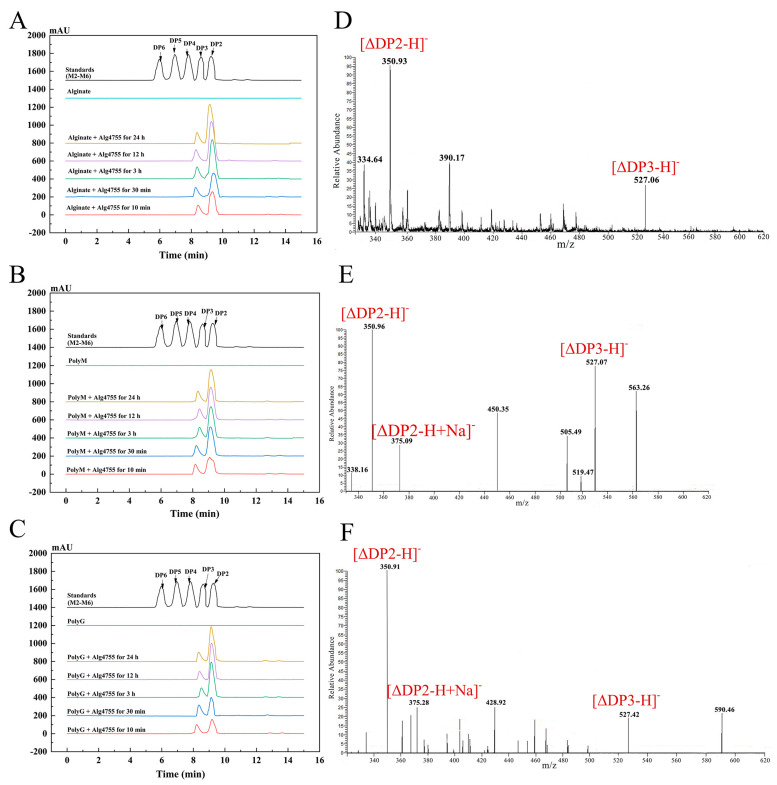
HPLC analysis of 0–24 h products of Alg4755 with (**A**) sodium alginate, (**B**) polyM, and (**C**) polyG. The degradation products were analyzed by monitoring at 210 nm using a UV detector. Saturated mannuronate oligosaccharides from DP2 to DP6 were taken as the standards. Analysis of the 24 h product composition of Alg4755 by ESI-MS with (**D**) sodium alginate, (**E**) polyM, and (**F**) polyG as substrates.

The degradation products of Alg4755 were analyzed by high-performance liquid chromatography (HPLC) over a time gradient of 0–24 h (Figure 7A–C). The final products of sodium alginate, polyM, and polyG were AOS containing disaccharides (DP2) and trisaccharides (DP3), which indicated that Alg4755 was an endolytic lyase. During the initial stage of the reaction, the three substrates were primarily broken down into disaccharides, with a small amount of trisaccharides. As the reaction progressed, the accumulation of disaccharides increased while the amount of trisaccharides remained relatively constant. To further verify the composition of the degradation products, the degradation products were analyzed by electrospray ionization mass spectrometry (ESI-MS) (Figure 7D–F). In the negative ion mode, the results are similar to those of HPLC. The degradation products were mainly alginate disaccharides with small amounts of alginate trisaccharides (the degrees of polymerization were represented by mass-to-charge ratios of products, with DP2 and 3 at 351 and 527 *m*/*z*, respectively). This result is similar to most other alginate lyases of the PL7 family, which produce oligosaccharides with DP of 2–5 mainly in an endolytic manner [31,43].

### 2.8. Three-Dimensional Structure Analysis of Alg4755

**Figure 8:**
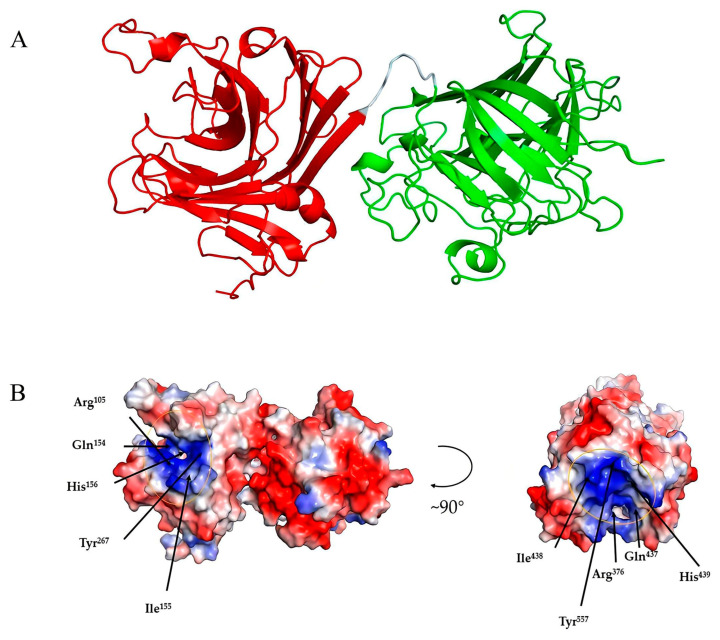
Three-dimensional structure analysis of Alg4755. (**A**) Predicted three-dimensional structure of Alg4755 using AlphaFold2. The predicted Alg4755 has two structural domains. The red region (left) is at the N-terminal (residues 57 to 286) and the green region (right) is at the C-terminal (residues 295 to 582). The cyan area is an octapeptide linker. (**B**) Electrostatic surface analysis of the entire structure of Alg4755. The residues in both structural domains that potentially play a key role in the catalytic process are located within the positively charged gaps, marked by yellow circles. The blue color in the diagram indicates a positive charge and the red color indicates a negative point.

Among the crystallized PL7 alginate lyases, Alg4755 showed the highest identity (45.61%) with that of AlyC3 (PDB code: 7C8G) from Psychromonas sp. The 3D structure of Alg4755 was predicted by AlphaFold2 [44]. The structural quality of Alg4755 was assessed using the SAVES v6.0 online server (https://saves.mbi.ucla.edu/, accessed on 10 April 2023). Ramachandran plots indicated that 85% of residues were in the most favored region, with 11.6% in the additional allowed region, 2.3% in the generously allowed region, and 1.1% in the disallowed region (Figure S4). The best model had an ERRAT (overall quality factor) test score of 95.43 (Figure S5) and a Verify3D test result of 93.31% (Figure S6). Therefore, the predicted 3D structure of Alg4755 was reasonable. The results show that Alg4755 consisted of two structural domains of the Alginate_lyase2 superfamily with an octapeptide as a linker (Figure 8A). The N-terminal structural domain had amino acid residues ranging from 57 to 286 and the C-terminal structural domain had amino acid residues ranging from 295 to 582. The Alg4755 protein had two structural domains that adopted a β-jelly roll fold, which was commonly found in alginate lyases from families PL7, 14, 18, and 36 [45,46]. Each domain contained four helices and two large β-sheets, namely Sheet A (SA) and Sheet B (SB). Sheet A consisted of nine β-strands, while Sheet B consisted of seven β-strands, and all of them were arranged in an antiparallel manner. These β-strands collectively form the main structure of the enzyme.

Alginate cleavage is a process that involves acid-base catalysis of the β-elimination reaction. This reaction takes place between the +1 site and −1 site of the alginate chain, and requires neutralization of the negative charge on the +1 site carboxylic group [26]. According to the sequence comparison with other PL7 family alginate lyases, His^156^, Arg^105^, Tyr^267^, and Gln^154^ amino acid residues in the N-terminal structural domain are strictly conserved in PL7 alginate lyase, and His^439^, Arg^376^, Tyr^557^, and Gln^437^ amino acid residues in the C-terminal structural domain are also strictly conserved in PL7 alginate lyase (Figure 2C). Previous studies also indicated that the above amino acids were the key residues for the alginate β-elimination reaction, such as PL7 cleavage enzymes A1-IÍ, FlAlyA, and AlyA1PL7 [47,48,49]. Previous studies proposed that arginine and glutamine could neutralize the negative charge on the carboxyl group, while histidine was used to remove the C-5 proton. Furthermore, tyrosine could donate a proton to cleave the 1,4-linkage, resulting in the formation of a double bond [39,50,51]. In addition, the electrostatic surface analysis showed that residues Arg^105^, Gln^154^, His^156^, and Tyr^267^ in the N-terminal structural domain were located within positively charged gaps, as well as the C-terminal structural domain (Figure 8B). This might form a binding pocket for the negatively charged alginate substrate.

## 3. Materials and Methods

### 3.1. Materials and Strains

Strain S10 was isolated from the intestine of *Apostichopus japonicus* in Changdao county, Shandong province, China. It was preserved by the Shandong Marine Resource and Environment Research Institute; pET-28a (+) and *E. coli* BL21(DE3) were purchased from Shanghai Sangon Biotech Co., (Shanghai, China); polyM and polyG (6–8 kDa, purity ≥ 98%) were purchased from Qingdao Zhibo Biotechnology Co., (Qingdao, China); and other chemical reagents were purchased from Sinopharm (Beijing, China).

### 3.2. Screening and Identification of Strain S10

The gut suspension of sea cucumber was mixed with sodium phosphate-buffered salt (PBS) solution (pH 7.4) at a ratio of 1:10 (*v*/*v*). The diluted solutions were spread onto the solid medium (yeast extract 0.1%, peptone 0.5%, agar 2%, and NaCl 3%) containing sodium alginate (0.3%), followed by incubating statically at 28 °C for 2–3 days. Furthermore, the plates were stained with Gram’s iodine solutions. Single colonies with hyaline circles were selected and cultured. The seed cultures (3%, *v*/*v*) were then inoculated into the fermentation medium (alginate 0.3%, yeast extract 0.1%, peptone 0.5%, and NaCl 3%) and cultured at 28 °C with shaking (180 rpm) for 48 h. Moreover, the fermentation liquids were centrifugated at 8000 rpm for 10 min, and then the supernatants were used to determine the enzymatic activity of alginate lyases. The strain with the highest activity was named S10, which was used for further study.

Strain S10 was incubated on solid medium for 24 h. The cells were then collected by scraping them into a centrifuge tube and then washed three times with PBS buffer (pH 7.4). Subsequently, the cells were fixed with 2.5% pentanediol for 3 h, followed by washing with PBS buffer three times to remove the remaining pentanediol. To dehydrate strain S10, six concentration gradients of ethanol solutions (20%, 40%, 60%, 70%, 80%, and 100%) were used for 15 min each. Moreover, the samples were immersed in a mixture (tert-butanol/ethanol, 1/1, *v*/*v*) for 15 min before being stored in tert-butanol. The treated samples were added dropwise to the coverslips, after which the coverslips were pre-cooled at −80 °C. Finally, the samples were imaged using a scanning electron microscope (JSM-6380LV, JEOL, Tokyo, Japan).

The strain S10 was sent to Shanghai Meiji Biomedical Technology Co., Ltd. (Shanghai, China) for 16S rRNA gene sequencing using primers 1492R (GGTTACCTTGTTACGACTT) and 27F (AGAGTTTGATCCTGGCTCAG) [52]. The sequencing results were analyzed using the EzbioCloud (http://www.ezbiocloud.net/; accessed on 10 April 2023) server and NCBI database BLAST (https://blast.ncbi.nlm.nih.gov/; accessed on 10 April 2023) online program (Sequence ID: LD944400.1). The neighbor-joining phylogenetic tree was constructed using MEGA11 software (version 11.0.13) with bootstrap repeating 1000 times.

### 3.3. Complete Genome Sequencing of Strain S10

The genome of strain S10 was extracted using the Bacterial Genome Extraction Kit (Solarbio, Beijing, China). For whole genome sequencing of strain S10, the Illumina HiSeq 1500 system was used in combination with the PacBio RS II system. The scan map of the strain S10 genome was assembled using the short sequence assembly software SOAPdenovo (version 2.04) to splice multiple K-mer parameters on the optimized sequences after second generation sequencing to obtain the optimal contig assembly results. The reads were then compared to the contigs, and the assembly results were partially assembled and optimized to form scaffolds based on the paired-end and overlap relationships of the reads. After quality control, we performed a trigeneration sequence assembly using the compliant clean data and the assembly software Unicycler (version 0.4.9) [53]. During the assembly process, we utilized the Pilon [54] software (version 1.22) for sequence correction. Finally, the corrected sequencing data were assembled into gap-free overlapping clusters. The integrity of the genome was assessed using BUSCO [55] software. The coding sequences (CDS) of S10 were predicted using GeneMarkS [35] software (version 4.30). Gene islands and prophages were predicted using IslandPath-DIOMB (version 1.0.0) and PhiSpy [56] software. tRNA genes, rRNA genes, and other non-coding RNA genes were predicted using the tRNAscan-SE [57] software (version 1.3.1), RNAmmer [58] software (version 1.2), and the Rfam database [59], respectively. To visualize the genomic information, genomic circle maps were generated using Circos [36] software (version 0.69.6).

### 3.4. Bioinformatics Analysis and Structure Prediction of Alg4755

Putative alginate lyases in strain S10 were predicted using the dbCAN meta server (https://bcb.unl.edu/dbCAN2/blast.php; accessed on 10 April 2023) [60]. The Alg4755 sequence was analyzed by BLAST (https://blast.ncbi.nlm.nih.gov/; accessed on 10 April 2023) and its corresponding amino acid sequence was obtained using the Expasy website (https://web.expasy.org/translate/; accessed on 10 April 2023). The SPs were predicted using the SignalP 5.0 server (http://www.cbs.dtu.dk/services/SignalP/; accessed on 10 April 2023) [61]. The theoretical PI and MW were predicted using the Expasy Server (https://web.expasy.org/protparam/; accessed on 10 April 2023) [62]. Domain analysis was performed by blasting against the Conserved Domain Database (https://www.ncbi.nlm.nih.gov/Structure/cdd/wrpsb.cgi; accessed on 10 April 2023) [63]. MEGA 11 software was used to construct the phylogenetic tree. Multiple sequence alignments were exported using ESPript 3.0 (http://espript.ibcp.fr/ESPript/ESPript/; accessed on 10 April 2023). Moreover, the protein structure of Alg4755 was predicted and evaluated using AlphaFold2 (London, UK) [44] and SAVES v6.0 online server (https://saves.mbi.ucla.edu/; accessed on 10 April 2023), respectively.

### 3.5. Cloning, Expression, and Purification of Recombinant Alg4755

The gene sequence without the termination codon of Alg4755 was optimized and synthesized by Shanghai Sango Biotech Co., Ltd. (Shanghai, China), which was further ligated to the pET-28a (+) plasmid with *Nde*I-*Xho*I restriction sites. The resulting recombinant plasmid was finally transformed into *E. coli* BL21(DE3) for expression. Thereafter, the recombinant strain was first incubated in LB liquid medium (+30 μg/mL kanamycin) at 37 °C with shaking overnight (180 rpm). The seed liquid was then inoculated into 300 mL LB medium (+30 μg/mL kanamycin) and incubated at 37 °C until OD_600_ was about 0.8, followed by the addition of IPTG solutions with a final concentration of 0.5 mM. The medium was then incubated at 18 °C with shaking (180 rpm) for 6 h to induce the expression of Alg4755. Induced cells were collected by centrifugation and resuspended in 30 mL bacterial protein preparation lysate (Sangon Biotech, Shanghai, China). After treatment with sonification, the supernatant and pellet of the cell lysis were separated by centrifugation at 4° C for 30 min. Moreover, the expressed Alg4755 with 6 × His tag was purified using a Ni-NTA column (Cowin Biotech, Jiangsu, China). The purified Alg4755 solutions were transferred to a dialysis bag with a cut-off molecular weight of 15 kDa and dialyzed with the complexation buffer (50 mM Tris-HCl, pH 8.0, 5 mM reduced glutathione, 1 mM oxidized glutathione, and 10 mM EDTA-2Na) at 4 °C for 6 h to remove imidazole and other metal ions. Finally, the dialysis bags were transferred to 50 mM Tris-HCl (pH 8.0) buffer for dialysis overnight, after which the proteins were further analyzed by SDS-PAGE.

### 3.6. Determination of the Enzymatic Activity and Protein Content

Enzymatic activity was determined using the DNS method. First, 100 uL of the enzyme solution was mixed with 100 μL of 0.3% (*w*/*v*) sodium alginate solution and incubated at 35 °C for 30 min. Then, 300 μL of DNS was added into the reaction medium, followed by boiling for 5 min. After adding 1 mL of distilled water and cooling, the absorbance at 520 nm was measured using an enzyme-labeled instrument (Thermo Fisher Scientific, Shanghai, China). The amount of reducing sugars was calculated using glucose as a standard. One unit of alginate lyase activity (U) was defined as the amount of enzyme required to produce 1 µg of reducing sugar per minute.

The protein concentration was determined using the Lowry method protein concentration determination kit (Solarbio, Beijing, China) using bovine serum albumin as a standard.

### 3.7. Enzyme Characterization of Alg4755

The optimal pH and temperature of Alg4755 were determined using 0.3% sodium alginate as a substrate at different pH (6–10.5) and different temperatures (25–60 °C), respectively. The thermal stability was determined by incubating Alg4755 at different temperatures ranging from 25 to 60 °C for 2 h, and the residual activity was then measured. Similarly, the pH stability was assessed by measuring residual activity after incubating Alg4755 in different buffers (50 mM PBS buffer, 50 mM Tris-HCl buffer, and 50 mM Glycine-NaOH buffer) with pH ranging from 6 to 10.5 for 2 h.

The effect of different metal ions on the activity of recombinant enzyme Alg4755 was studied. The reaction mixtures (100 μL of purified enzyme solution and 100 μL of 0.3% sodium alginate solution mixture, 100 µL of purified enzyme solution containing 1 mg of purified enzyme) were individually added with different metal ions (AlCl_3_, CoCl_2_, MnCl_2_, MgCl_2_, CuSO_4_, FeCl_2_, FeCl_3_, NaCl, CaCl_2_, LiCl, BaCl_2_, and NiCl_2_) to a final concentration of 1 mM, reacted under optimal conditions for 30 min, and the enzyme activity was measured. The effect of inhibitors and detergents on the activity of Alg4755 was investigated. Two different concentrations of inhibitor and decontaminant were added to the purified enzyme solution, then mixed with sodium alginate solution, and reacted at 35 °C for 30 min. Subsequently, the enzyme activity was measured.

To investigate the substrate specificity, purified Alg4755 was individually added to the reaction media containing 0.3% sodium alginate, polyM, and polyG, which was further incubated under optimal reaction conditions for 30 min. The kinetic parameters (K_m_, K_cat_ and V_max_) of Alg4755 toward three substrates were studied by measuring the enzymatic activity of Alg4755 under different concentration of substrates (1–5 mg/mL).

### 3.8. Action Pattern and Product Analysis of Alg4755

HPLC with Superdex peptide 10/300 GE Colum (GE Healthcare, Pittsburgh, PA, USA) at 210 nm was used to separate and monitor the products. The reaction mixtures (400 µL) containing 100 µL of purified Alg4755 (containing 1 mg purified enzyme) and 300 µL of substrates (0.3% *w*/*v* sodium alginate, 0.3% *w*/*v* polyM, and 0.3% *w*/*v* polyG, respectively) were incubated at 35 °C for 0–24 h. The samples were taken after reaction for 0 min, 10 min, 30 min, 3 h, 12 h, and 24 h. The mixture was eluted with 0.2 M NH_4_HCO_3_ at a flow rate of 0.3 mL/min. The hydrolysis products were further determined using ESI-MS. The conditions were as follows: the ion source power was 4.5 kV, the capillary temperature was from 275 to 300 °C, tube lens voltages was 250 V, the flow rate of sheath gas was kept at 30 arbitrary units, and the scanning range was from 150 to 1000 (*m*/*z*).

## 4. Conclusions

In this study, we isolated an alginate-degrading strain S10 from the gut of sea cucumber and determined the complete genome sequence. An alginate lyase named Alg4755 was identified, heterologously expressed, and characterized. Alg4755 belonged to the alginate lyase PL7-6 subfamily with two catalytic domains. The optimal temperature and pH of Alg4755 were 35 °C and pH 8.0, respectively. Furthermore, Alg4755 showed higher thermal and pH stability when compared with reported alginate lyase. More importantly, the main products of Alg4755 were determined as alginate disaccharides with a small amount of alginate trisaccharides, which could significantly simplify downstream purification procedures. The above results indicate that Alg4755 had desirable catalytic properties for alginate disaccharide and trisaccharide production, which could promote the development of alginate-related industries.

## Figures and Tables

**Table 1:** Purification of recombinant alginate lyase Alg4755.

Purification Step	Total Volume (mL)	Total Protein (mg)	Total Activity (U)	Specific Activity (U/mg)	Purification	Yield (%)
Crude enzyme	30.0	22.51	2639.70	117.27	1.0	100.0
Purified enzyme	6.0	2.26	2173.98	961.94	8.2	82.4

**Table 2:** Specific activities and kinetic parameters of Alg4755 toward sodium alginate, polyM, and polyG (data shown are the mean ± SD, *n* = 3).

Substrate	Sodium Alginate	PolyM	PolyG
Activity (U/mg)	961.94 ± 6.4	891.20 ± 4.9	1026.37 ± 7.2
K_m_(mM)	5.41 ± 0.38	3.64 ± 0.24	1.28 ± 0.15
V_max_ (mol/s)	0.21 ± 0.17	0.39 ± 0.08	1.13 ± 0.14
k_cat_ (s^−1^)	3.82 ± 0.28	7.09 ± 0.52	20.55 ± 0.96
k_cat_/K_m_ (s^−1^/mM)	0.71 ± 0.06	1.95 ± 0.04	16.05 ± 0.77

## Data Availability

The 16S rRNA gene sequence of strain S10 has been submitted to the NCBI GenBank database under accession number OL944400.1. It can be found here: (https://www.ncbi.nlm.nih.gov/nuccore/OL944400.1/, accessed on 10 April 2023). The raw genomic data of strain S10 has been submitted to the NCBI Sequence Read Archive (SRA) database under accession number SRR26253840. It can be found here: (https://www.ncbi.nlm.nih.gov/sra/SRR26253840/, accessed on 10 April 2023). The genome-assembly data for strain S10 have been submitted to the NCBI GenBank database under accession number CP135968.1-CP135970.1. The amino acid sequence of Alg4755 has been submitted to the NCBI GenBank database under the accession number OQ884715.1. It can be found here: (https://www.ncbi.nlm.nih.gov/nuccore/OQ884715.1/, accessed on 10 April 2023).

## Supplementary Materials

The following supporting information can be downloaded at: https://www.mdpi.com/article/10.3390/md21110564/s1, Table S1: Genome statistics of strain S10; Figure S1: Circos map of strain S10 chromosome 1; Figure S2: Circos map of strain S10 chromosome 2; Figure S3: Circos map of strain S10 plasmid A; Figure S4: Ramachandran plot of the predicted protein structure of Alg4755; Figure S5: ERRAT detection results of Alg4755 predicted protein structure; Figure S6: Verify3D detection results of Alg4755 predicted protein structure.
