# Supplementary material for: Genome Sequencing-Based Mining and Characterization of a Novel Alginate Lyase from Vibrio alginolyticus S10 for Specific Production of Disaccharides
Source: Mar Drugs. 2023 Oct 27;21(11):564. doi: 10.3390/md21110564 (PMC10672080; doi:10.3390/md21110564)
Supplement: Supplementary file 1 [file marinedrugs-21-00564-s001.zip › marinedrugs-2560855-supplementary.pdf]

Supplementary Materials

Genome Sequencing-Based Mining and Characterization of a Novel Alginate Lyase from *Vibrio alginolyticus* S10 for Specific Production of Disaccharides

Zhiqiang Shu [1,2,†], Gongming Wang [2,3,†], Fang Liu [2,3], Yingjiang Xu [2,3], Jianan Sun [4,5], Yang Hu [4,5], Hao Dong [4,5,*] and Jian Zhang [2,3,*]

1 Department of Food Science and Technology, Shanghai Ocean University, Shanghai 200120, China; s13970318519@163.com

2 Shandong Marine Resource and Environment Research Institute, Yantai 264006, China; wgmsd105@163.com

3 Yantai Key Laboratory of Quality and Safety Control and Deep Processing of Marine Food, Yantai 264006, China

4 Qingdao Key Laboratory of Food Biotechnology, College of Food Science and Engineering, Ocean University of China, Qingdao 266404, China

5 Key Laboratory of Biological Processing of Aquatic Products, China National Light Industry, Qingdao 266404, China

* Correspondence: donghao@ouc.edu.cn (H.D.); zjsd408@163.com (J.Z.)

† These authors contributed equally to this work.

Content

Table S1

Figure S1, S2, S3, S4, S5 and S6.

Table S1. Genome statistics of strain S10

Type	Length(bp)	Amount
Whole gene sequence	5,397,046	---
GC content	44.59%	---
Total length of CDS	4,587,936	4,936
Total transposon length	---	7
Short scattered sequence	---	41
Long scattered sequence	---	36
Gene island	---	12
tRNA	---	127
rRNA	8,592	57

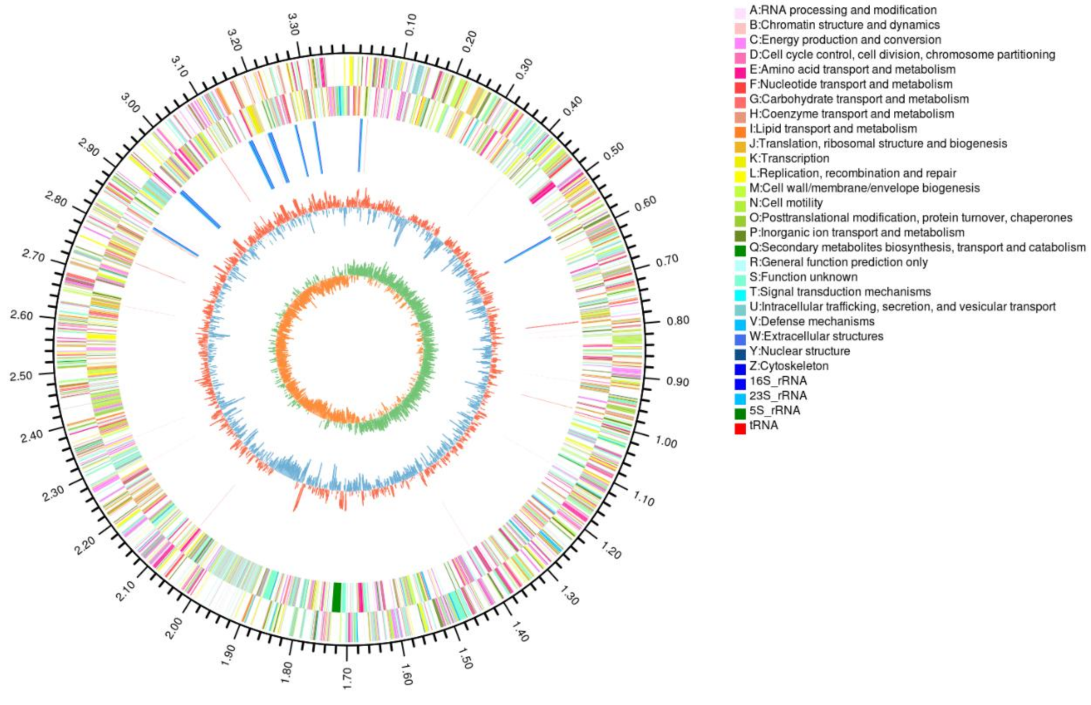

Figure S1. Circos map of strain S10 chromosome 1.

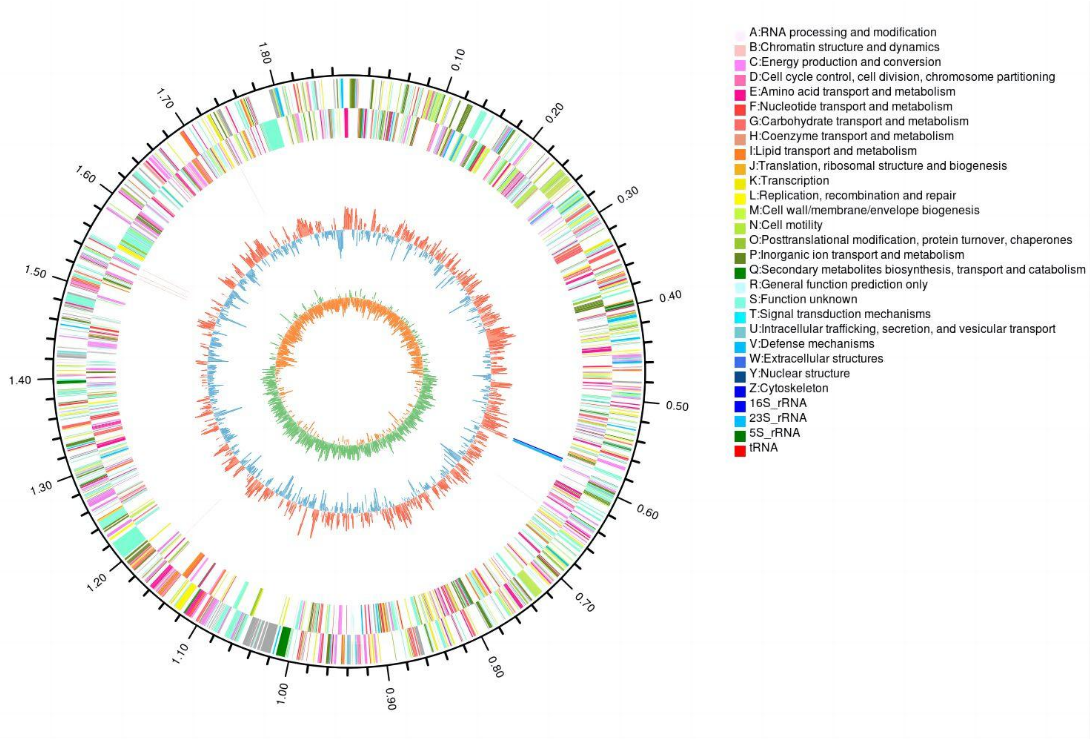

Figure S2. Circos map of strain S10 chromosome 2.

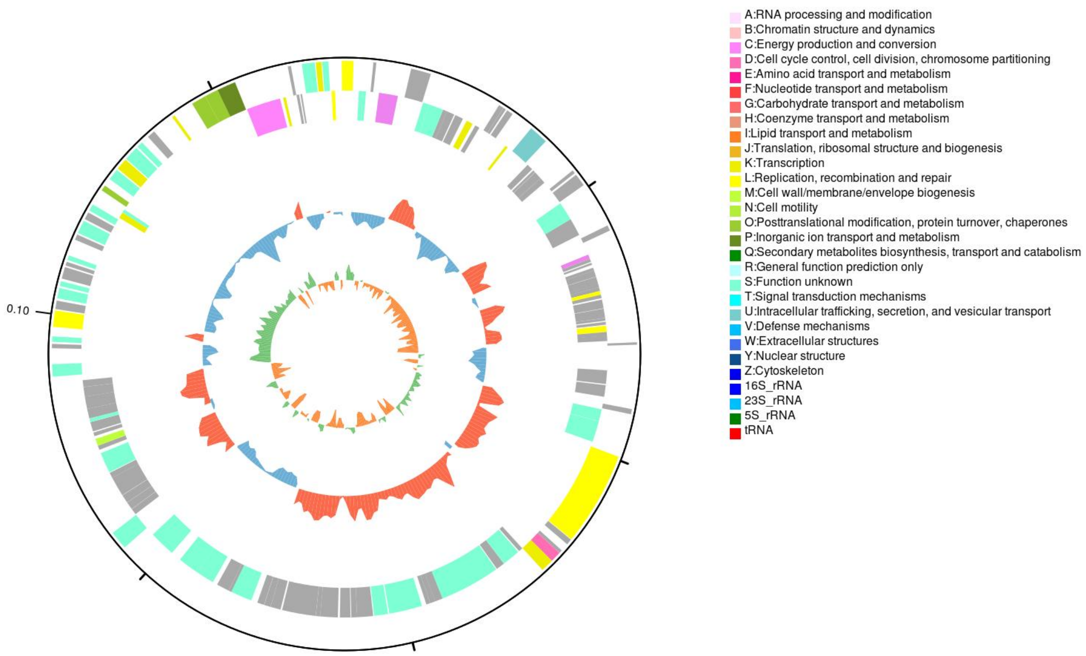

Figure S3. Circos map of strain S10 plasmid A.

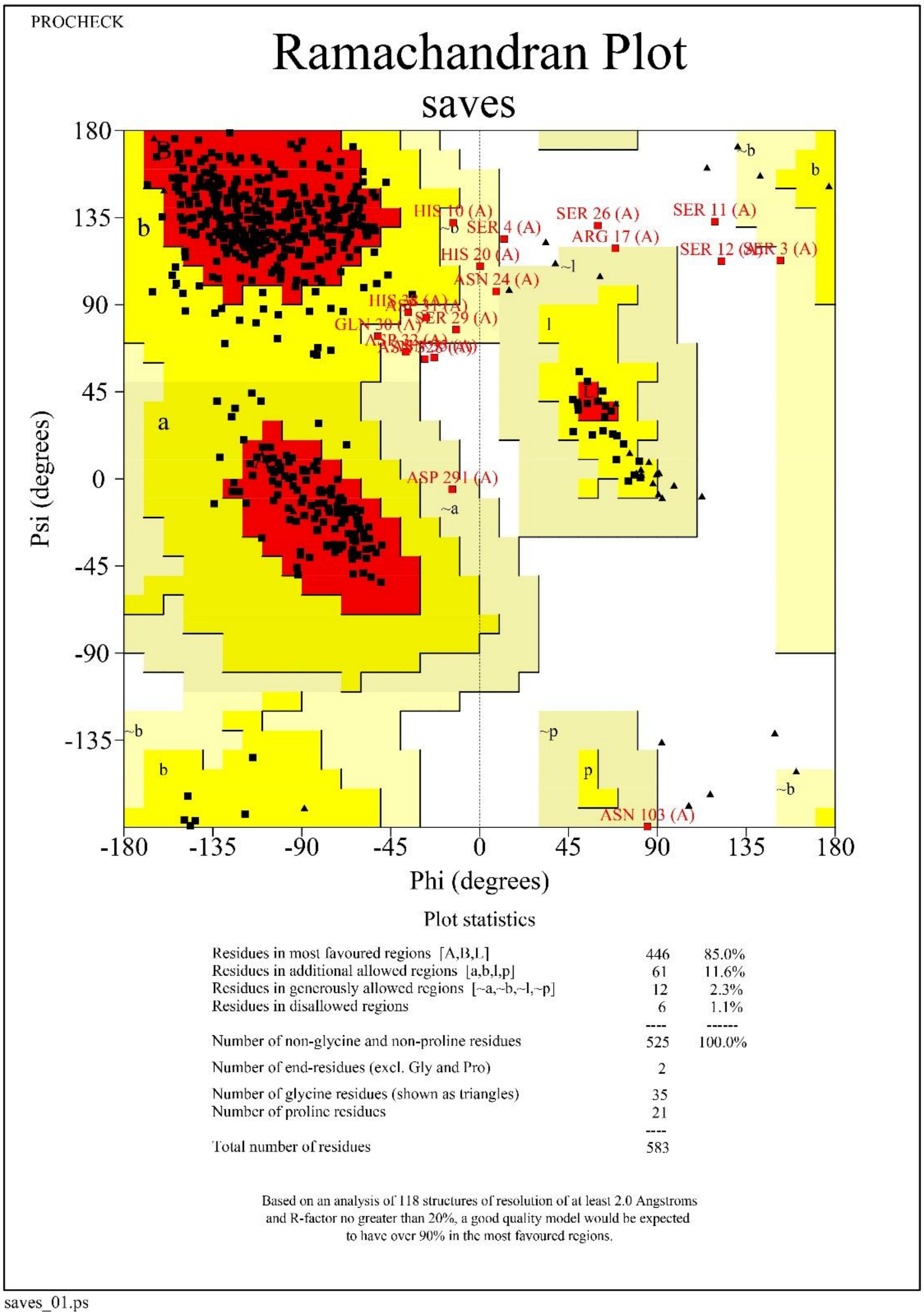

Figure S4. Ramachandran plot of the predicted protein structure of Alg4755

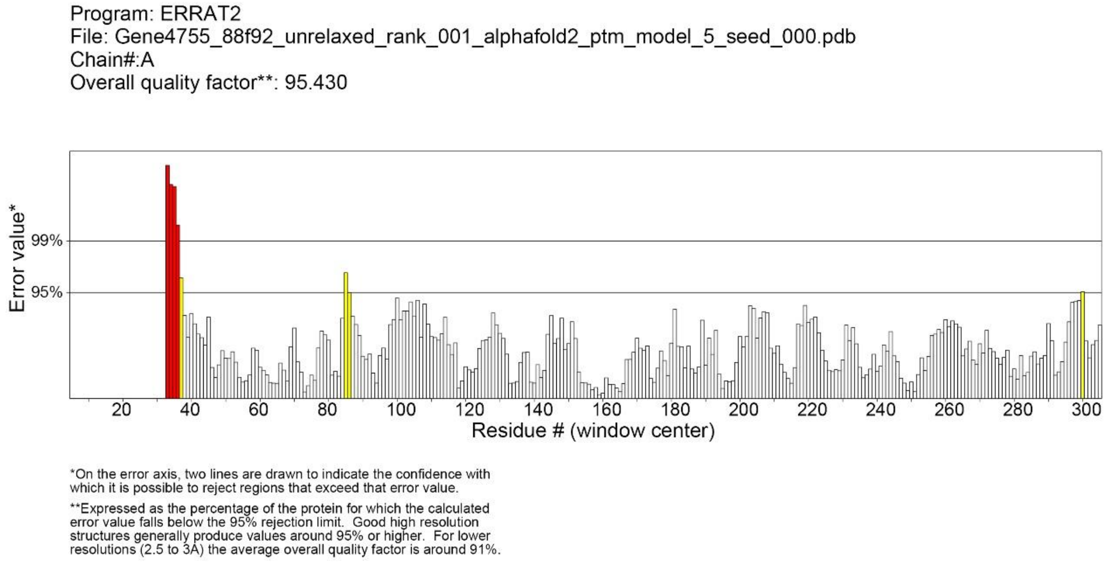

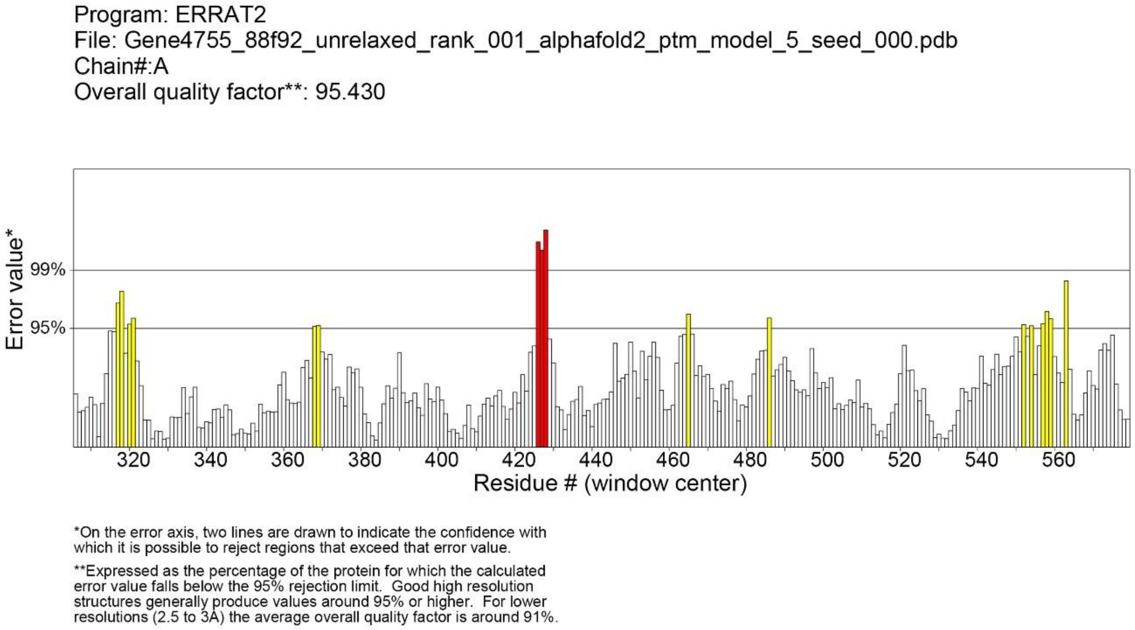

Figure S5. ERRAT detection results of Alg4755 predicted protein structure.

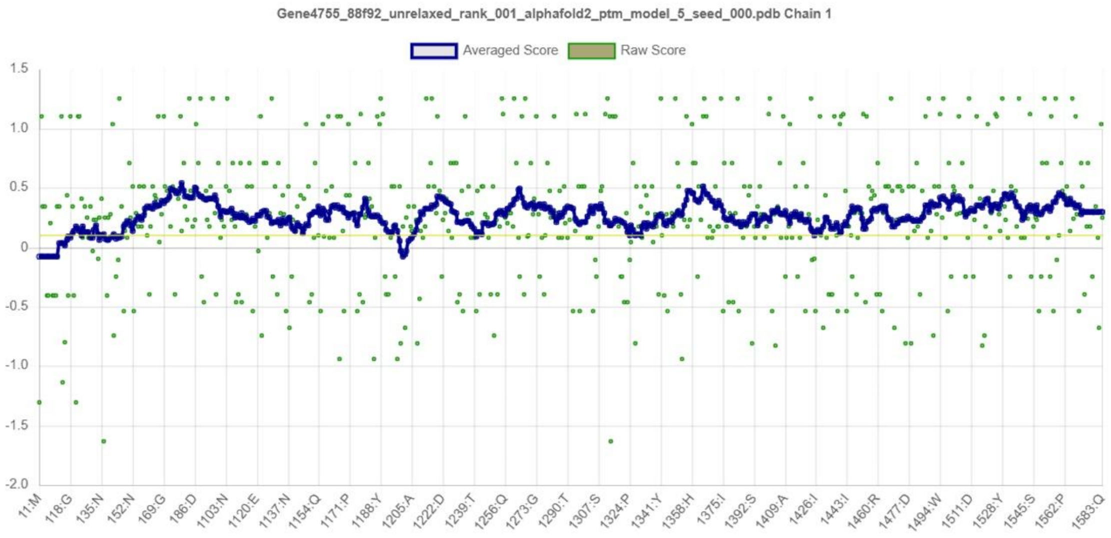

Figure S6. Verify3D detection results of Alg4755 predicted protein structure.
